# Supplementary material for: Prediction, syntax and semantic grounding in the brain and large language models
Source: Sci Rep. 2026 Mar 10;16:8728. doi: 10.1038/s41598-026-41532-0 (PMC12979642; doi:10.1038/s41598-026-41532-0)
Supplement: Supplementary file 1 — Supplementary Information. [file 41598_2026_41532_MOESM1_ESM.pdf]

Supplementary Material of Paper "Prediction, Syntax and Semantic Grounding in the Brain and Large Language Models"

NIKOLA KÖLBL[1,2], STEFAN RAMPP[3,4,5], MARTIN KALTENHÄUSER[3], KONSTANTIN TZIRIDIS [1], ANDREAS MAIER[6], THOMAS KINFE[7,9], RICARDO CHAVARRIAGA[8], PATRICK KRAUSS[1,2,7,9,+], ACHIM SCHILLING[1,2,7,9,+,*]

[1]Neuroscience Lab, University Hospital Erlangen, Germany
[2]CCN Group, Pattern Recognition Lab, FAU Erlangen-Nürnberg, Germany
[3]Department of Neurosurgery, University Hospital Erlangen, Germany
[4]Department of Neuroradiology, University Hospital Erlangen, Germany
[5]Department of Neurosurgery, University Hospital Halle (Saale), Germany
[6]Pattern Recognition Lab, FAU Erlangen-Nürnberg, Germany
[7]Neuromodulation and Neuroprosthetics, University Hospital Mannheim, University Heidelberg, Germany
[8]ZHAW Zürich, Switzerland
[9]BGU Ludwigshafen, Germany
[+]both authors contributed equally
[*]corresponding author

Additional figures are presented in this section, offering deeper insights into the EEG and MEG analyses. Suppl. Fig. 1 illustrates an example of average baseline activity, generated using randomly selected time points with an equivalent number of trials as those for nouns. The signal amplitudes for ERFs (left) and ERPs (right) are much lower compared to those for word-class-specific ERFs and ERPs in Figure 2 and 3.

Suppl. Figures 2 (ERFs) and 3 (ERPs) depict significant clusters identified through a cluster-based paired t-test, comparing word-class ERFs and ERPs against the baseline. The poorer source reconstruction for EEG data can be demonstrated by the lack of differences in source-space activations for nouns and verbs in Suppl. Figure 4 compared to Figure 7.

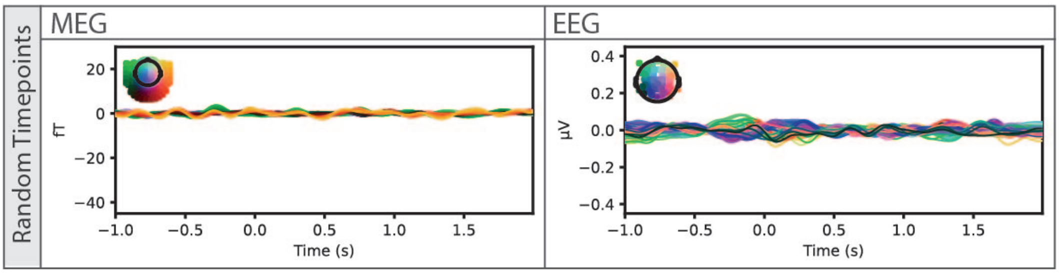

Suppl. Fig 1: Grand average ERFs (left) and ERPs (right) of brain activity at randomly chosen time points. Example shows the signals for the same amount of trials as for the nouns.

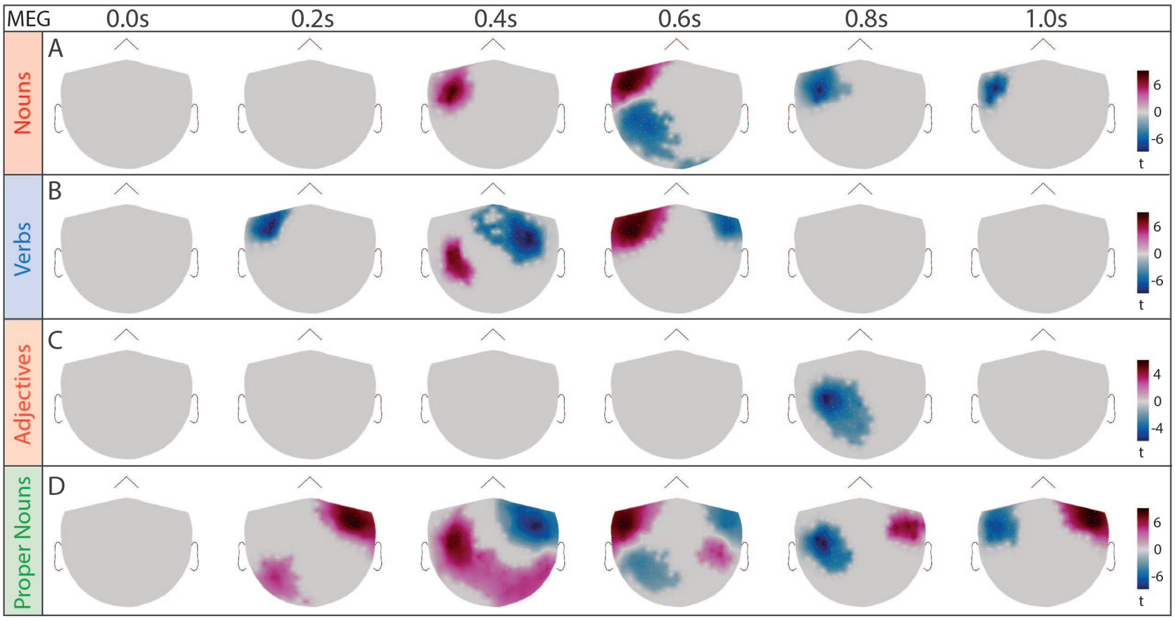

Suppl. Fig 2: Significant differences of spatial distribution of ERFs (topomaps) between word classes (nouns, verbs, adjectives, proper nouns) and baseline based on cluster-based paired t-test (time interval: 0.0-1.0 s, shown time points: 0.0 s, 0.2 s, 0.4 s, 0.6 s, 0.8 s, 1.0 s). A) nouns: red: p=0.018, blue: p=0.00004. B) verbs: red: p=0.0328, blue: p=0.0008. C) adjectives: red: p=0.0488, D) proper nouns: red: p=0.0004, blue right hemisphere: p=0.0120, blue left hemisphere: p=0.0020.

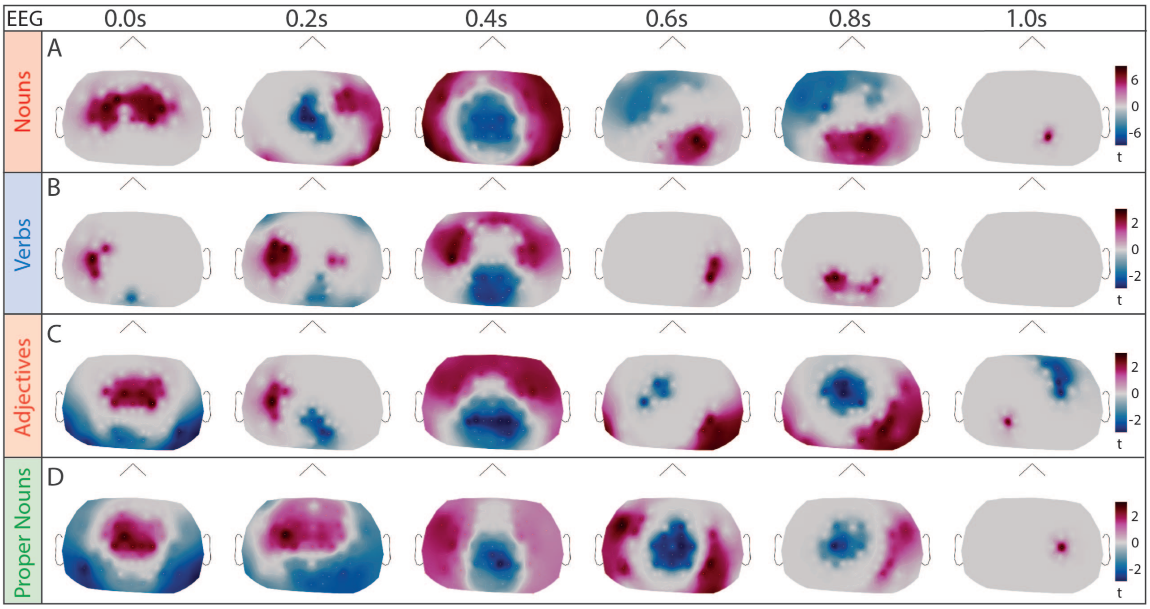

Suppl. Fig 3: Significant differences of spatial distribution of ERPs (topomaps) between word classes (nouns, verbs, adjectives, proper nouns) and baseline based on cluster-based paired t-test (time interval: 0.0-1.0 s, shown time points: 0.0 s, 0.2 s, 0.4 s, 0.6 s, 0.8 s, 1.0 s). A) nouns: red: p=0.0004, blue: p=0.0004. B) verbs: red: p=0.0004, blue: p=0.0028. C) adjectives: red: p=0.0004, blue: p=0.0004. D) proper nouns: red: p=0.0004, blue: p=0.0004.

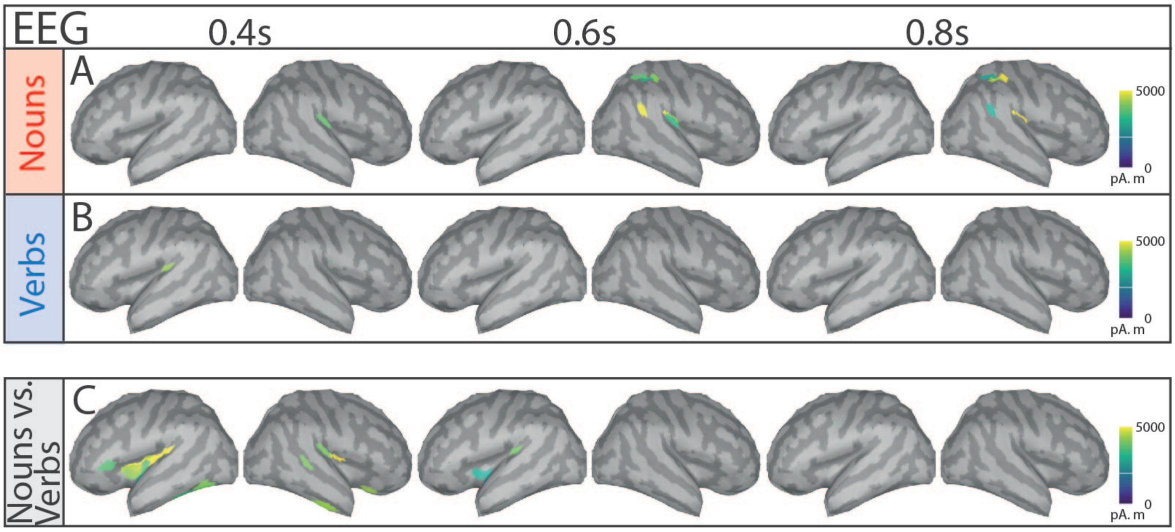

Suppl. Fig 4: Significant differences of source space activity between nouns and baseline, verbs and baseline, and nouns and verbs based on cluster-based paired t-test (shown time points: 0.4 s, 0.6 s, 0.8 s). Shown amplitudes is simulated MEG activity in pAm using the resulting t-values of significant regions ($p<0.05$). A: Noun-ERPs vs. random-time point ERPs (significant difference from baseline), B: Verb-ERPs vs. random time point ERPs and C) Noun-ERPs vs. verb-ERPs.
